# A Mutation in the *MYBL2-1* Gene Is Associated with Purple Pigmentation in *Brassica oleracea*

**DOI:** 10.3390/ijms231911865

**Published:** 2022-10-06

**Authors:** Emil Khusnutdinov, Alexander Artyukhin, Yuliya Sharifyanova, Elena V. Mikhaylova

**Affiliations:** Institute of Biochemistry and Genetics Ufa Federal Research Center RAS, Prospekt Oktyabrya 71, 450054 Ufa, Russia

**Keywords:** *Brassicaceae*, *Brassica oleracea*, DFR, *MYBL2*, SNP, RNA, CRISPR

## Abstract

Anthocyanins are well-known antioxidants that are beneficial for plants and consumers. Dihydroflavonol-4-reductase (*DFR*) is a key gene of anthocyanin biosynthesis, controlled by multiple transcription factors. Its expression can be enhanced by mutations in the negative regulator of anthocyanin biosynthesis myeloblastosis family transcription factor-like 2 (*MYBL2*). The expression profiles of the *DFR* gene were examined in 43 purple and green varieties of *Brassica oleracea* L., *Brassica napus* L., *Brassica juncea* L., and *Brassica rapa* L. *MYBL2* gene expression was significantly reduced in purple varieties of *B. oleracea*, and green varieties of *B. juncea*. The *MYBL2* gene sequences were screened for mutations that can affect pigmentation. Expression of the *DFR* gene was cultivar-specific, but in general it correlated with anthocyanin content and was higher in purple plants. Two single nucleotide polymorphysms (SNPs) were found at the beginning of the DNA-binding domain of *MYBL2* gene in all purple varieties of *B. oleracea*. This mutation, leading to an amino acid substitution and the formation of a mononucleotide repeat (A)_8_, significantly affects RNA structure. No other noteworthy mutations were found in the *MYBL2* gene in green varieties of *B. oleracea* and other studied species. These results bring new insights into the regulation of anthocyanin biosynthesis in genus *Brassica* and provide opportunities for generation of new purple varieties with precise mutations introduced via genetic engineering and CRISPR/Cas.

## 1. Introduction

The genus *Brassica* includes species distributed worldwide, such as *B. oleracea*, *B. napus*, *B. juncea*, and *B. rapa*. *Brassica* vegetables are economically important dietary products rich in phenolic compounds, carotenoids, vitamins C, and E [1]. Purple varieties of *Brassica* sp. are the most rare and beneficial for health due to the presence of anthocyanins. These phenolic compounds can be used as natural dyes and colorants in food due to the high thermal stability [2]. Anthocyanins are also associated with increased stress tolerance [3].

The anthocyanin biosynthesis pathway is well-studied; however, the specific genes responsible for purple pigmentation in *Brassica* sp. have not been discovered yet. The genetic regulation of anthocyanin accumulation is traditionally studied in ornamental plants, such as petunia, phalaenopsis, and snapdragon [4,5,6,7].

The key gene of anthocyanin biosynthesis, encoding dihydroflavonol-4-reductase (*DFR*), is controlled by multiple transcription factors and micro RNAs. Low expression of this gene, which catalyzes the synthesis of leucoanthocyanidin, is suggested to be the bottleneck, preventing anthocyanin synthesis in plants [8]. PAP1 (AN2), PAP2, TT8, TTG1, MYB2, and Delila are known as its positive regulators. GLABRA2, HAT1, CPC, MYBL2, MYB1, and MYB57 were reported to downregulate the expression of the *DFR* gene. Three types of transcription factors: MYB, MYC/basic Helix-Loop-Helix (bHLH), and WD40-repeat (WDR) proteins usually act in MBW complexes. These complexes bind to the DFR promoter and regulate the transcription of *DFR* gene to alter anthocyanin biosynthesis [9].

In phalaenopsis, *DFR* and *MYB* transcripts were present in the purple rather than the white sectors of the flower; however, the expression level of other structural and regulatory genes was the same in both sectors [4]. Zoysiagrass cultivars with green and purple spikes and stolons differed by the expression level of *Anthocyanidin synthase* and *DFR* genes [10]. In black-skinned pomegranate, the *DFR* gene expression was higher, and *AN2* gene expression was lower than in samples with white skin [11]. In the white petunia, the introduction of an exogenous *DFR* gene resulted in a pink-flowered phenotype [12]. In general, in white flowers the expression of structural anthocyanin genes is undetectable [4,13]. However green varieties of *Brassica* sp. produce some anthocyanins, their content is by several orders of magnitude lower than in purple varieties [14]. Unlike petunia, which has three different *DFR* genes to control pigmentation in different organs, most *Brassicaceae* have only one functional gene and usually accumulate anthocyanins in all tissues [15]. Overexpression of the exogenous *AtDFR* gene in *B. napus* increased *DFR* transcript levels, anthocyanin accumulation in the shoots, and salt tolerance [3]. Purple pigmentation may appear or decrease in response to stress, lighting intensity, changes in nitrogen concentration and other factors. This suggests that in green *Brassica* sp. *DFR* expression is not blocked absolutely, but is controlled via a quantitative regulatory system [16].

Some structural and regulatory genes of *Brassicaceae* can be solely responsible for purple pigmentation or its absence [17]. Mutations such as indels can affect anthocyanin pigmentation. A mutation in *GLABRA3* gene contributed to a 98% decrease in the *DFR* transcript content and anthocyaninless phenotype in *Arabidopsis thaliana* [8]. Insertion of a natural transposon in a *TT8* gene of *B. rapa* contributed to the loss of pigmentation in the seeds [14]. Mutation of the promoter activated the expression of the *BoMYB2* gene in purple cauliflower [18].

Mutations identified in the coding sequence of the *DFR* gene itself also can disrupt the functions of the protein [19,20]. Deletion of the *DFR* gene in purple ornamental kale resulted in a loss of pigmentation [21].

The negative regulator, *MYBL2*, which encodes a R3-MYB-related protein, appears to make the greatest contribution to changes in *DFR* gene expression and anthocyanin pigmentation in many plant species. *MYBL2* expression appears to be dependent on light intensity and temperature [22,23]. The MYBL2 transcription factor has a DNA-binding MYB domain and a repressive domain. In *A. thaliana* the minimal repression domain of MYBL2 consists of six amino acids (TLLLFR) at the carboxyl terminus [24]. Possible inhibition mechanisms of this negative regulator have not yet been clarified. It either binds to the bHLH protein and prevents the formation of MBW complexes or replaces it in the complex [25,26,27].

It has been confirmed that silencing of the *MYBL2* gene in *A. thaliana* promoted *DFR* expression [26,27]. Unlike *DFR* gene, *MYBL2* might have several orthologs in *Brassica* sp. [28]. For example, *MYBL2-1* mRNA was not detectable in purple *B. oleracea*; however *MYBL2-2* was expressed. This suggests that only *MYBL2-1* is closely associated with anthocyanin production in *B. oleracea*; however, the second ortholog probably did not retain this function. Anthocyanin hyperaccumulation in these plants resulted from either the promoter substitution or deletion of the *MYBL2-1* gene [25]. Data on the role of *MYBL2-1* gene in *B. rapa* are contradictory. It has been suggested that a 100-bp insertion in the third exon of *MYBL2-1* gene is associated with purple pigmentation in this species [17,29]. However, *MYBL2-1* gene is highly expressed in another purple variety of *B. rapa* and therefore may serve as a positive regulator [30].

However it is clear that the *MYBL2* gene plays a key role in the regulation of anthocyanin biosynthesis, data on mutations that reliably induce anthocyanin accumulation in genus *Brassica* are very limited. Most of the experiments were carried out on the model plant *A. thaliana*, which has a single *MYBL2* gene copy. The studied *B. oleracea* and *B. rapa* varieties were of Asian origin; however, purple *Brassica* vegetables are supposed to be native to the Mediterranean region of Europe [29]. In *B. napus* and *B. juncea* this gene has never been studied. *MYBL2* has not yet been subjected to CRISPR/Cas editing, although it appears to be a perfect target gene, responsible for phenotypic changes [9].

To figure out if any mutations in the *MYBL2* gene can be associated with pigmentation in 43 purple and green varieties of *B. oleracea* L., *B. napus* L., *B. juncea* L., and *B. rapa* L., expression profiles of *DFR* and *MYBL2* genes and sequencing of the *MYBL2* gene were performed. Anthocyanin content was measured in green and purple varieties. The possibilities to introduce precise mutations to the *MYBL2* gene via CRISPR/Cas editing were evaluated.

## 2. Results

### 2.1. Expression Profiles of the DFR Gene in Brassicaceae

**Figure 1:**
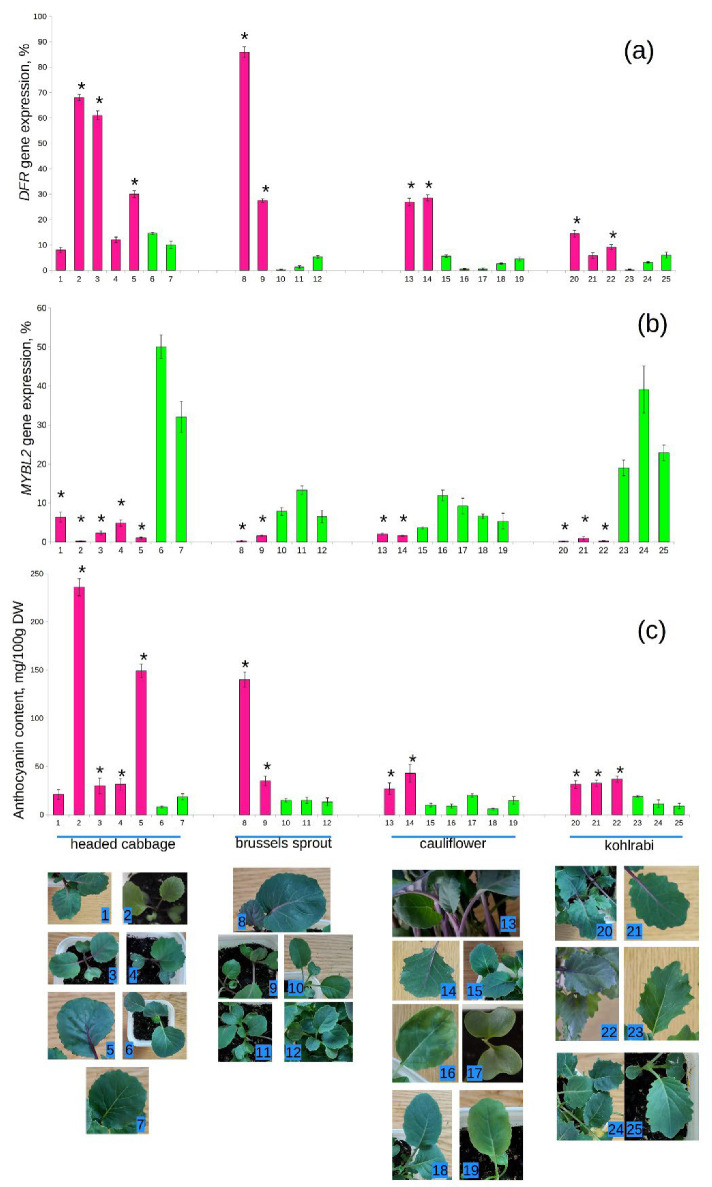
Relative expression level of the *DFR* gene (**a**), *MYBL2* gene (**b**), and anthocyanin content (**c**) in purple and green cultivars of *B. oleracea*. Visible pigmentation is shown in the photographs. Cultivars are numbered as follows: 1—Kalibos; 2—Ludmila; 3—Ruby; 4—Mars; 5—Firebird; 6—Royal Vantage; 7—Moscow Late; 8—Garnet bracelet; 9—Bunch of grapes; 10—Rosella; 11—Hercules; 12—Sapphire; 13—Gardener’s dream; 14—Purple ball; 15—Bird’s milk; 16—Baby; 17—Flame star; 18—Green bunch; 19—Alpha; 20—Madonna; 21—Violetta; 22—Vienna purple; 23—Vienna white; 24—Gulliver; 25—Picante. Varieties declared as purple are highlighted in pink. Asterisk (*) indicates a significant difference from unpigmented plants of the same cultivar.

Visible purple pigmentation in *B. oleracea* was not directly related to the expression of the *DFR* gene (Figure 1). In general, purple varieties of the same cultivar produced more mRNA of the *DFR* gene than green ones, but there was a great difference between cultivars. Purple kohlrabi (Figure 1(20–22)) did not differ significantly from several green varieties of headed cabbage by the expression of *DFR* gene (Figure 1(6,7)); however, the phenotypic differences were clearly visible. The primary leaf veins, petioles, and stems were the most pigmented organs in *B. oleracea*, and not the leaf lamina.

In several varieties of headed cabbage and Brussels sprout (Figure 1(2,3,8)) the *DFR* gene was expressed at the level of the reference gene (70–90%); however, in cauliflower and kohlrabi it only reached 20–30% of the reference gene expression. Our results indicate that the *DFR* gene is not decisive for the formation of anthocyanin pigmentation in all varieties of cabbage; therefore, other genes may be involved.

**Figure 2:**
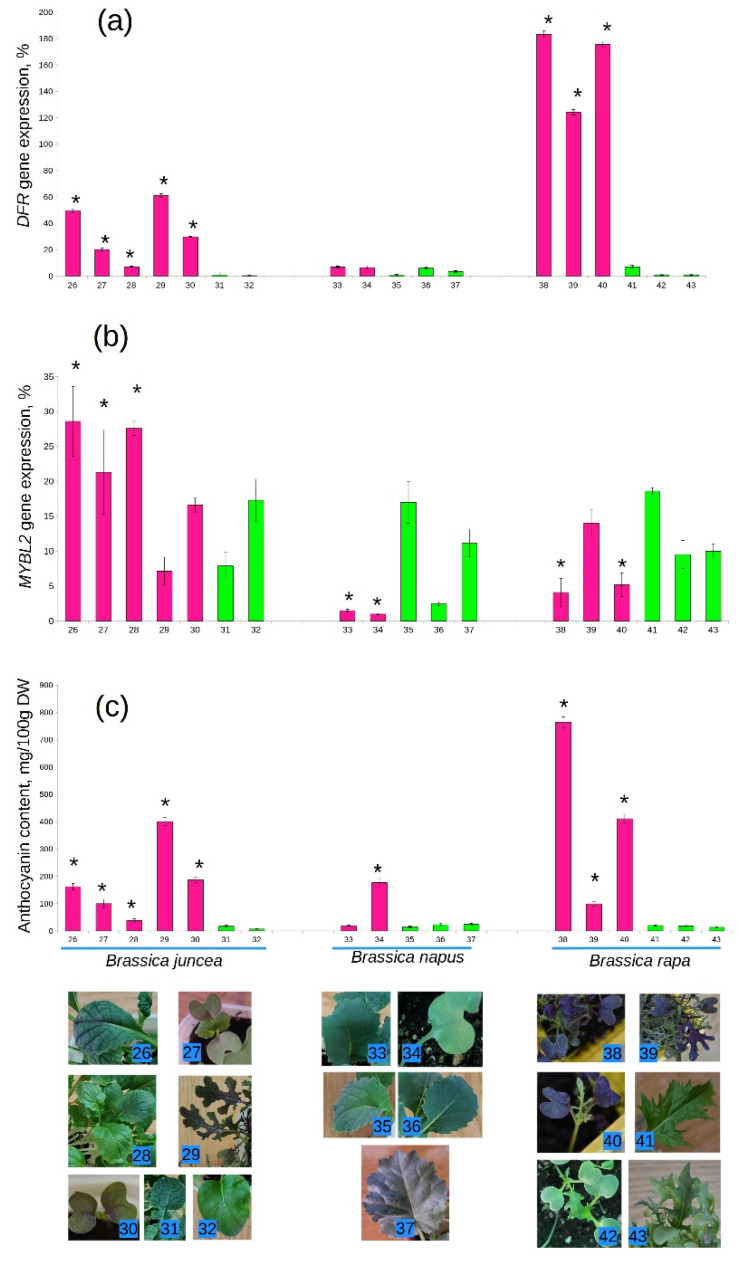
Relative expression level of the *DFR* gene (**a**), *MYBL2* gene (**b**) and anthocyanin content (**c**) in purple and green cultivars of *B. juncea*, *B. napus*, and *B. rapa*. Visible pigmentation is shown in the photographs. Cultivars are numbered as follows: 26—Vitamin; 27—Red velvet; 28—Freckle; 29—Miracles in the sieve; 30—Red giant; 31—Wavelet; 32—Vigorous; 33—rutabaga Krasnoselskaya; 34—rutabaga Gera; 35—rutabaga Novgorodskaya; 36—rutabaga Child love; 37—fodder cabbage Veha; 38—Ruby little mermaid; 39—Mizuna Red; 40—Mizuna purple; 41—Impulse; 42—The little mermaid; 43—Mizuna green. Varieties declared as purple are highlighted in pink. Asterisk (*) indicates a significant difference from unpigmented plants of the same species.

*B. rapa* was the only species where mRNA content of the *DFR* gene was higher than those of the reference gene (Figure 2(38–40)). The cotyledons of *B. rapa* were also the most pigmented among all studied *Brassica* species. On the contrary, *B. napus* with pigmented roots had green leaves with unremarkable expression level of the *DFR* gene (Figure 2(33,34)). One of the *B. juncea* varieties was supposed to accumulate anthocyanins (Figure 2(28)); however, its leaves were not visibly pigmented and demonstrated low expression level of the *DFR* gene, but not as low as in green varieties. In green cabbage this gene was expressed at a certain level (Figure 1); however, in green *B. juncea* and *B. rapa* its mRNA content was close to zero (Figure 2(31,32,42,43)).

Our results indicate that expression profiles of the *DFR* gene in *Brassicaceae* are species-specific and even cultivar-specific.

### 2.2. Expression Profiles of the MYBL2 Gene in Brassicaceae

However *DFR* gene expression in several purple varieties of *B. oleracea* was no higher than in green ones, *MYBL2* mRNA content was very low and did not exceed 10% in all purple varieties (Figure 1(1,4,21). In green varieties of *B. oleracea MYBL2* gene expression was also cultivar-specific. In headed cabbage and kohlrabi it was the highest (40–50% of the reference gene), but in Brussels sprout and cauliflower it was almost as low as in purple plants (Figure 1(8–19)). Nevertheless, in these two cultivars a strong negative correlation was observed between the expression levels of *DFR* and *MYBL2* genes (correlation coefficients = −0.78 and −0.8, respectively); however, in headed cabbage and kohlrabi coefficients were lower (−0.5 and −0.65).

Interestingly, in three purple varieties of *B. juncea*, the expression of *MYBL2* gene was significantly higher than in green varieties (Figure 2(26–28)), which was unexpected of a putative negative regulator of anthocyanin biosynthesis. No correlation was found between the expression levels of *DFR* and *MYBL2* genes in this species.

The content of *MYBL2* mRNA in purple *B. rapa* and *B. oleracea* was comparable, except for the Mizuna Red cultivar (Figure 2(39)). The level of *DFR* expression in this cultivar was also lower than in Ruby little mermaid and Mizuna purple (Figure 2(38,40)). The correlation coefficient between the expression levels of the two studied genes in *B. rapa* was −0.62.

Unexpectedly, the correlation between the expression levels of *DFR* and *MYBL2* genes appeared to be the strongest in *B. napus* (−0.99), which were not visibly pigmented.

### 2.3. Total Anthocyanin Content

To reveal how *DFR* and *MYBL2* gene expression levels affect the accumulation of anthocyanins, their content was measured in green and purple varieties.

The content of anthocyanins in the studied plants ranged from 6 to 411 mg/100 g of dry weight (DW). In general, this parameter positively correlated with the *DFR* gene expression level (correlation coefficient = 0.83). Among groups, only in *B. napus* and headed cabbage correlation between *DFR* mRNA and anthocyanin content was weaker (0.4 and 0.66). Correlation with *MYBL2* gene expression was negative, but very weak. It was notable only in kohlrabi (−0.92), Brussels sprout (−0.69), and *B. rapa* (−0.75). These results are indicative of the direct involvement of the *DFR* gene in anthocyanin biosynthesis and regulatory role of the *MYBL2* gene, which may differ depending on the plant variety and exogenous factors.

Green varieties contained on average 14 mg/100 g of anthocyanins, while in purple varieties anthocyanin content was 10 times higher. These results are consistent with the literature data [10]. However, there were some exceptions. For example, in the rutabaga Gera (Figure 2(34)), which was supposed to have pigmentation only in roots, the leaves contained no less anthocyanins than the purple varieties of *B. oleracea*. The expression of the *DFR* gene in *B. napus* was also higher than in most of the anthocyaninless varieties of other species. This indicates that the regulation of anthocyanin biosynthesis in *B. napus* may be more complex due to ploidy and multiple copies of the gene.

In purple-headed cabbages Kalibos and Mars (Figure 1(1,4)) *DFR* mRNA content was the same as in green varieties. Indeed, anthocyanin content in these plants was also low, and *MYBL2* expression was higher than in other purple varieties of *B. oleracea*, but much lower than in green-headed cabbages.

The highest anthocyanin content as well as the highest *DFR* gene expression was observed in *B. rapa*. As far as these species have thin, delicate leaves, they may contain less debris and more anthocyanins per unit weight. The involvement of the *MYBL2* gene in anthocyanin biosynthesis in these species is very likely. On the contrary, no noticeable effect of the *MYBL2* expression on the accumulation of anthocyanins was observed in *B. juncea*.

To determine if the negative regulator of anthocyanin biosynthesis MYBL2 is related to the *DFR* expression profiles and anthocyanin content, we subjected the samples of each species and cultivar to sequencing.

### 2.4. Sequencing of the MYBL2 Gene

However it was earlier reported that purple varieties of *B. oleracea* lacked *BoMYBL2-1* coding sequences [25], amplification was successful in all studied samples. A 100-bp insertion, associated with purple pigmentation in *B. rapa* [29], also was not detected.

**Figure 3:**
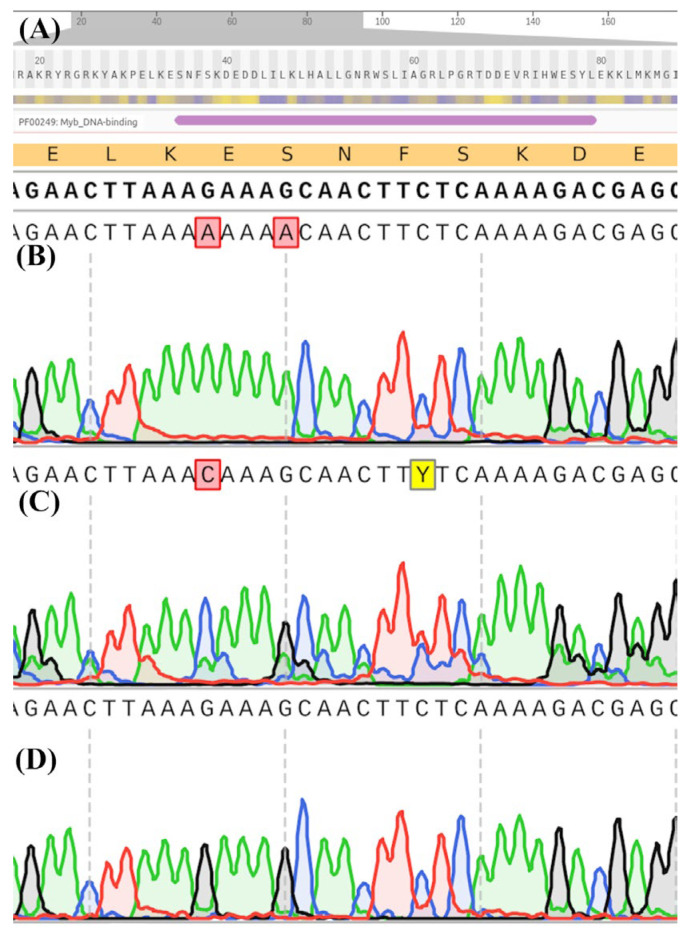
The localization of a DNA-binding domain in a *MYBL2* gene (**A**) and the sequence of the beginning of this domain in *B. oleracea* varieties Gardener’s dream (**B**), Gulliver (**C**), and green *B. juncea* variety Vigorous (**D**).

We discovered two completely new SNPs in a *MYBL2* sequence of all analyzed purple varieties of *B. oleracea*, which affected the beginning of the DNA-binding MYB domain, resulting in the replacement of two amino acids (from KESN to KKNN), compared with *B. rapa* (Figure 3). The mutation was common for all cultivars of *B. oleracea*; however, it was not detected in any of the green varieties, or in *B. juncea*, *B. napus*, and *B. rapa*. In green varieties of *B. oleracea* there was also one SNP in this site, compared with the *MYBL2* gene of *B. rapa*, resulting in an amino acid change (GAA into CAA – KESN into KQSN); however, it was outside of the DNA-binding domain. Therefore, the mutation probably did not affect DNA binding. Other species did not have any mutations within this site. Close to the carboxyl terminus there was also one SNP, specific for purple cabbage (GTG into GTT); however, it did not result in an amino acid substitution.

It should be noted that there were occasional heterozygous mutations in green varieties of cabbage (Figure 3C); however, in purple varieties there were almost none which means they have homozygous alleles of *MYBL2* gene.

There were other differences between the varieties, which cannot be directly associated with anthocyanin pigmentation. For example, a 30 bp long insertion (GTAATGTGAATGTTATTTTTTTTGCAAAAA) was observed in the noncoding sequence of the *MYBL2* gene in purple and green *B. oleracea*, compared with *B. rapa*.

Therefore, we were unable to detect any noteworthy mutations in purple varieties of *B. juncea*, *B. napus*, and *B. rapa*, which can be associated with hyperpigmentation.

## 3. Discussion

As a result of a present study, we report a mutation in the beginning of a DNA-binding site of a *MYBL2* gene of purple *B. oleracea* for the first time. According to the NCBI database, AAAGAAAGCAAC into AAAAAAAACAAC (KESN into KKNN) mutation is unique and has never previously been found. The presence of the same mutation in all purple varieties of *B. oleracea* strongly suggests that it is directly associated with anthocyanin pigmentation.

There are several possible mechanisms by which this mutation can result in phenotypic changes. First of all, it is a mononucleotide (A)_8_ repeat (SSR) in the coding region, which can lead to gene inactivation, changes in function and phenotype [31]. SSRs can serve as binding sites for regulatory elements, may form altered DNA secondary structures, and affect gene translation. For example, translational repression of the *MYBL2* gene by MiR858 was shown to enhance anthocyanin biosynthesis in seedlings of *A. thaliana* [32]. SSRs often cause DNA slippage during DNA replication [33]. This mutation also creates the KKXX dilysine motif, which promotes the relocation of proteins from the Golgi apparatus to the endoplasmic reticulum [34].

**Figure 4:**
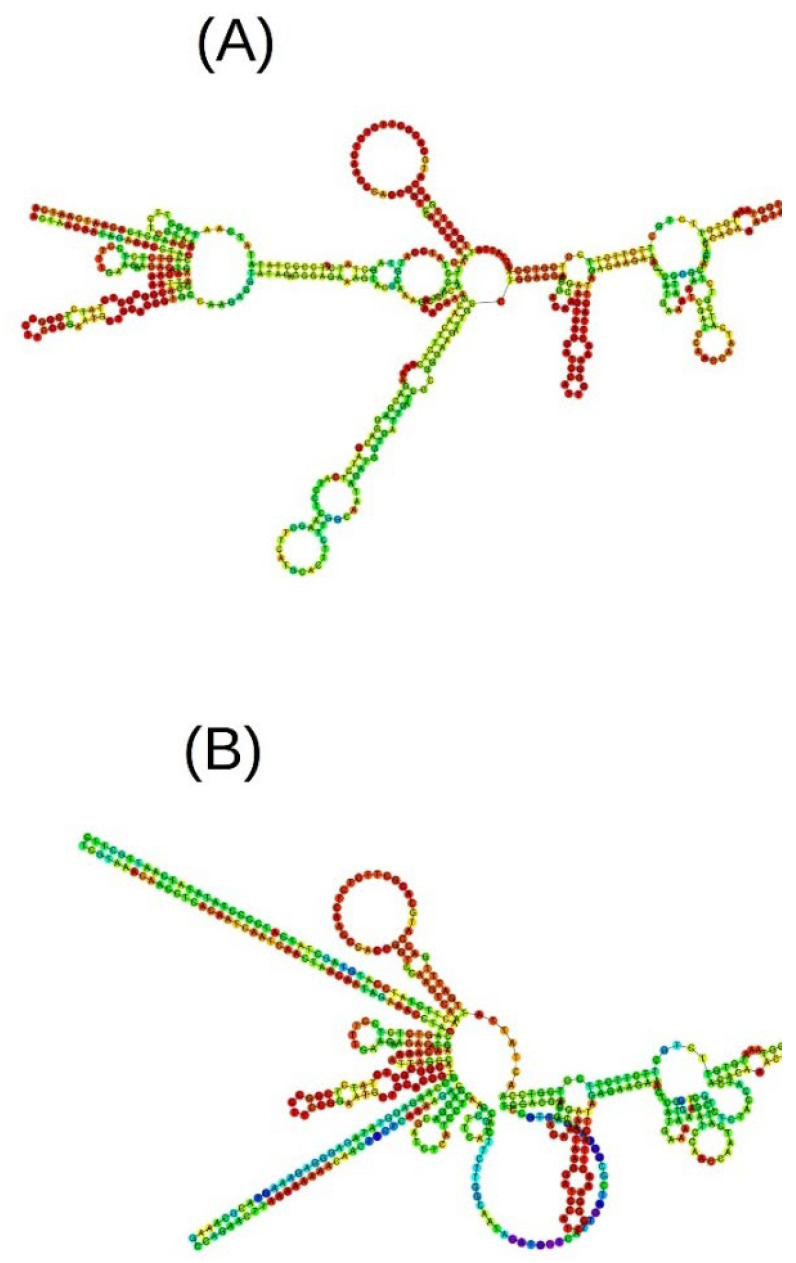
Part of a predicted RNA structure of the normal (**A**) and mutant (**B**) *MYBL2* gene.

The mutation affects the secondary structure of RNA (Figure 4), which plays a central role in post-transcriptional regulation of gene expression and affect recognition by ribosomes. U- and A-rich sites may correlate with the recognition of endonucleases for regulating alternative polyadenylation [35].

In all purple varieties of *B. oleracea* expression level of the *MYBL2* gene was lower than 10%; however, in green varieties it can be as high as 50% (Figure 1). Variability in the mRNA content of this gene was not so pronounced in other plant species (Figure 2). It is interesting that strong correlation between the expression of *MYBL2* and *DFR* genes was also observed in *B. napus*, which is indicative of the special role of *MYBL2* gene in the C genome. In *B. oleracea* and *B. napus* this gene negatively affected the expression of *DFR*, such as in *A. thaliana* [26,27].

No significant mutations were detected in the *MYBL2-1* gene sequence in purple varieties of *B. juncea*, *B. napus*, and *B. rapa*, which suggests that *DFR* gene expression in these species can be regulated differently. It has been shown that six amino acids (TLLLFR) at the carboxyl terminus are obligatory for the repression activity of the *MYBL2* transcription factor in *A. thaliana* [24]. However, in *B. rapa* there is a substitution of the last amino acid from R to Q (CAG). In *B. juncea MYBL2* expression was even higher in purple varieties, which suggests that, such as in *B. rapa*, this gene can also serve as a positive regulator of anthocyanin biosynthesis [30]. Therefore, in these species *MYBL2* may work through a different mechanism of repression activity or have none at all. In our study the reverse primer for gene amplification covered half of the sequence encoding this repression domain. Successful amplification indicated that in purple varieties of *B. rapa* and *B. juncea* it was present.

This also suggests that the absence of the PCR product of *MYBL2-1* gene in purple varieties of cabbage [25] may be due to primer design or contamination of the DNA with polyphenols, which can inhibit PCR reaction. There can be significant differences between European and Asian varieties of cabbage. All cultivars of *B. oleracea* involved in our study may have the same European (possibly Mediterranean) origin, because none of them were of Asian breeding. Some varieties, such as Kalibos, have been known in Europe for more than 200 years.

Interestingly, varieties of *B. oleracea*, such as Brussels sprout and kohlrabi, had different levels of *MYBL2* and *DFR* genes expression and anthocyanin content. In purple and green kohlrabi the difference between *MYBL2* mRNA content was dramatic; however, in green Brussels sprout *MYBL2* mRNA content was rather low. On the contrary, there was a great difference in *DFR* expression between green and purple cultivars of Brussels sprout. This may be explained by the fact that *DFR* is not the only gene controlled by MYBL2, and MYBL2 is not the only anthocyanin biosynthesis repressor in *Brassica*. In *B. oleracea* the content of anthocyanins increases with maturation, which indicates the important role of regulatory genes such as *MYBL2* in this process. The loss of *MYBL2* activity in *A. thaliana* resulted in an increase in mRNA content of leucoanthocyanidin dioxygenase (*LDOX)* and, to a lesser extent - flavanone 3β-hydroxylase (*F3H)* and chalcone isomerase *(CHI)* structural genes [22]. Similar to *DFR*, *LDOX* is regulated by MBW complexes [36]. LDOX is involved in anthocyanin biosynthesis pathway downstream of the DFR, promoting the transformation of leucocyanidin to cyanidin [37]. F3H and CHI are involved upstream and are able to provide more substrate for DFR.

It has been demonstrated that an insertion in the coding region of *LDOX* gene resulted in a complete loss of pigmentation in pomegranate [38]. In *A. thaliana* a single aminoacid substitution in LDOX resulted in a pale brown seed color and lack in anthocyanin accumulation [36]. *LDOX* gene of *Reaumuria trigyna* complemented reduced proanthocyanin and anthocyanin levels in the Arabidopsis loss of function mutant [39]. Transgenic *A. thaliana* overexpressing *RtLDOX2* accumulated more anthocyanins and flavonols only under abiotic stress, but also increased primary root length, biomass accumulation, and stress resistance [40]. There are several functional copies of endogenous *LDOX* gene in the genomes of *Brassica* sp., which complicates the investigation of their impact on the anthocyanin accumulation. Therefore, they remain understudied in these species. Consequently, *LDOX* may be a gene of interest in purple cabbage cultivars with low expression of *DFR* gene, such as kohlrabi.

We were unable to detect any significant mutations in *MYBL2-1* gene of *B. juncea*, *B. napus*, and *B. rapa*, including those previously described [25,28]. This suggests that other genes may be responsible for anthocyanin accumulation in studied varieties. There are a lot of candidate genes, including not only transcriptional repressors *CPC*, *LBD*, and *GLABRA2*, but also activators such as *PAP1*, *PAP2*, *MYB1*, and *MYB2* [9]. For example, a mutation in the promoter region activated the expression of *BoMYB2* gene in purple *B. oleracea* [18]. Whole genome sequencing might be a more suitable method for further studies of *B. juncea, B. napus*, and *B. rapa* purple varieties.

Knowledge of beneficial mutations can allow fast generation of new purple varieties via traditional selection, genetic engineering, and CRISPR/Cas. Precise editing in the mutation site of the *MYBL2-1* gene can allow to prove if two SNPs in the beginning of the DNA-binding domain are enough to induce athocyanin hyperaccumulation not only in *B. oleracea*, but also in other *Brassica* species.

**Figure 5:**
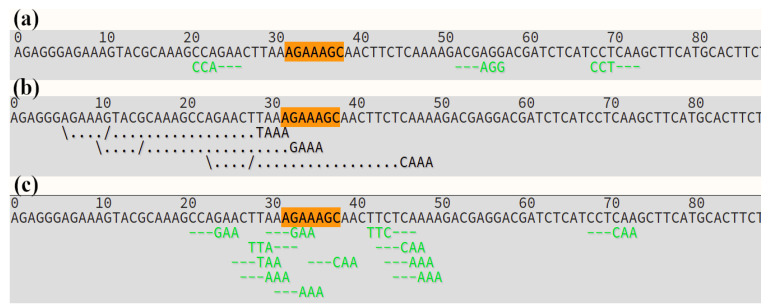
PAM sites in the beginning of the DNA-binding domain of a *MYBL2* gene, recognized by (**a**) Cas9, (**b**) Cas12a, (**c**) iSpyMacCas9.

To evaluate the possibility of CRISPR/Cas editing, we examined the mutation site in the beginning of the DNA-binding domain for the presence of the PAM sequences. There are several types of Cas nucleases, which recognize different PAM sequences [41]. The site of interest is AT-rich; therefore, the most common nuclease SpCas9 is poorly applicable (Figure 5a). Two of the three possible gRNAs close to the target site are inefficient, according to CRISPOR software calculations. The second most used nuclease Cas12a also is not appropriate for introducing the desired mutation, since the predicted double strand breaks are quite far from the target site (Figure 5b).

Modified nucleases such as iSpyMacCas9 [42], recognizing A-rich PAM sequence 5′-NAAR, can be a better choice to introduce desired mutations in the beginning of the DNA-binding domain of a *MYBL2* gene (Figure 5c).

Prime editing [43] might be the most suitable method to check if this particular mutation cause anthocyanin pigmentation in B. oleracea. With the use of elongated guide RNA, containing a donor sequence, it is possible to introduce the precise point mutations, identical to those in naturally bred purple cabbage. Nevertheless, according to the previous reports, many types of mutations in the MYBL2 gene, which are much easier to obtain, are applicable for generation of new purple varieties [25,26,27,29]. Any of the described nucleases can be used with multiple gRNAs to ensure complete excision of the target region or the whole gene. However complete excision may not be recommended as far as it can affect other functions of MYBL2-1 gene, such as trichome initiation and brassinosteroid signaling [26,44], absence of this gene copy did not seem to affect fitness of purple *B. oleracea* [25]. Therefore, attempts for introduction of the two desired SNPs are of little value to agriculture.

The discovery and introduction of point mutations, ensuring anthocyanin hyperaccumulation, can help to produce new purple varieties of economically important *Brassicaceae* crops. *Brassica* vegetables can be considered a functional food that can be used directly or as a dietary supplement [45]. Altering competition for substrate between flavonol synthase and dihydroflavonol-4-reductase by creating mutations in the *MYBL2* gene may also increase stress tolerance and nutritional value of new purple varieties.

## 4. Materials and Methods

### 4.1. Plant Materials

We analyzed 43 varieties of four economically important species of *Brassica*, including 22 varieties that were supposed to be purple, according to the manufacturer. *B. oleracea* was represented by 25 varieties, including purple-headed cabbage (Kalibos, Ludmila, Ruby, Mars, and Firebird), purple Brussels sprout (Garnet bracelet and Bunch of grapes), purple cauliflower (Gardener’s dream and Purple ball), and purple kohlrabi (Madonna, Violetta, and Vienna purple), as well as green varieties of headed cabbage (Royal Vantage and Moscow Late), Brussels sprout (Rosella, Hercules, and Sapphire), cauliflower (Bird’s milk, Baby, Flame star, Green bunch, and Alpha), and kohlrabi (Vienna white, Gulliver, and Picante). Anthocyanin pigmentation was observed in all varieties that were supposed to be purple, according to the manufacturer.

Five purple varieties of *B. juncea* (Vitamin, Red velvet, Freckle, Miracles in the sieve, and Red giant) and two green varieties (Wavelet and Vigorous) were used in this study. However, Freckle lacked anthocyanin pigmentation. Among five varieties of *B. napus*, rutabaga Krasnoselskaya and Gera were supposed to accumulate anthocyanins in roots; however rutabaga Novgorodskaya and Child love, as well as fodder cabbage Veha, were supposed to be anthocyaninless. Visible purple pigmentation was not detected in the leaves of any of these varieties. We also studied three purple varieties of *B. rapa* (Ruby little mermaid, Mizuna Red, and Mizuna purple) and three green varieties (Impulse, The little mermaid, and Mizuna green).

Green and purple varieties of each cultivar of *B. oleracea* and other studied species were subjected to Sanger sequencing of the *MYBL2* gene. Namely, green varieties Moscow late, Sapphire, Flame star, Gulliver, Vigorous, Novgorodskaya, Child love, and Impulse as well as purple varieties Ludmila, Garnet bracelet, Gardener’s dream, Purple ball, Madonna, Vienna purple, Vitamin, Krasnoselskaya, Gera, and Mizuna red were examined.

Plants were grown at 20 °C under 10,000 lux and a 16:8 h day:night photoperiod, generated by LED grow light. True leaves were used for anthocyanin content measurement. Other experiments were carried out on cotyledons, homogenized using FastPrep-24 bead beating grinder and lysis system (MP Biomedicals, Santa Ana, CA, USA).

### 4.2. Real-Time Quantitative PCR

RNA was extracted from 50 mg of the plant tissue using Lira reagent (Biolabmix, Novosibirsk, Russia). cDNA was synthesized with OT-M-MuLV-RH kit (Biolabmix, Novosibirsk, Russia) using oligo(dT) primer.

Real-time quantitative PCR (RT-qPCR) was performed on Quant studio 5 Real-Time PCR System (Thermo Fisher Scientific, USA) using BioMaster HS-qPCR Lo-ROX SYBR mix (Biolabmix, Novosibirsk, Russia) with the primers 5′-CGGATCTGCAGGTTTAACTG-3′ and 5′-GCCGACATGTGACTATCGG-3′ for the *DFR* gene and two primer pairs 5′-AGAGGGAGAAAGTACGCAAAG-3′ and 5′-CGGTTCGTCCAGGCAATCTT-3′; 5′-TTCATGCACTTCTTGGCAAT-3′ and 5′-CGGTTCGTCCAGGCAATCTT-3′ for the *MYBL2* gene. Primers were designed using NCBI Primer BLAST tool [46]. Actin7 (primers 5′-AGGAATCGCTGACCGTATGAG-3′ and 5′-GCTGAGGGATGCAAGGATGGA-3′) was used as a reference gene. This gene is stably expressed in normal conditions and under biotic and abiotic stress [47].

### 4.3. DNA Sequencing

DNA was extracted from plant tissue using CTAB method [48].

*MYBL2-1* gene was amplified using primers 5′-AGAGGGAGAAAGTACGCAAAG-3′ and 5′-AGAAGTGTTTCTTGACTCGTTGA-3′. PCR products were purified with ExoSAP-IT™ PCR Product Cleanup Reagent (Thermo Fisher Scientific, Waltham, MA, USA), prepared using BigDye™ Terminator v3.1 Cycle Sequencing Kit and subjected to Sanger sequencing via Applied Biosystems 3500 genetic analyzer (Thermo Fisher Scientific, Waltham, MA, USA).

### 4.4. Total Anthocyanin Content

Anthocyanin pigment concentration was measured by the spectrophotometric pH differential method using a Perkin Elmer LS 55 Luminescence Spectrometer (Perkin Elmer, Waltham, MA, USA) and expressed as cyanidin-3-glucoside equivalents. Anthocyanins in *Brassicaceae* are known to be cyanidin derivatives. Extracts from 15 mg of dried leaf tissue in pH 1.0 and pH 4.5 buffers were prepared and analyzed according to Lee et al. [49]. Measurements were performed in three technical and three biological replicates in 96-well plates.

### 4.5. Statistical Analysis

Three samples of each variety were used for DNA and RNA extraction. Primers were designed using NCBI Primer BLAST tool [46]. RT-qPCR was carried out for three replicates of each sample. Results of RT PCR were assessed by 2^−∆∆CT^ method. Sequencing was performed using forward and reverse primers for each sample. Sequences were aligned to the reference sequence of the *MYBL2-1* gene of *B. rapa* (JN379102) and analyzed using SnapGene software. Functional domains were analyzed via InterPro [50]. Search for gRNAs was performed in CRISPOR web tool [51]. RNA structure was predicted by RNAfold web tool [52]. For all experiments, means and standard deviation (*p* < 0.05) were compared by analysis of variance (ANOVA), and Pearson correlation coefficient was calculated using LibreOffice v.

## Data Availability

The sequence of the mutant genotype was submitted to the NCBI database (ON464161).
